# Functional assessments of foot strength: a comparative and repeatability study

**DOI:** 10.1186/s12891-019-2981-6

**Published:** 2019-12-14

**Authors:** Dustin A. Bruening, Sarah T. Ridge, Julia L. Jacobs, Mark T. Olsen, Dallin W. Griffin, Drew H. Ferguson, Kirk E. Bassett, A. Wayne Johnson

**Affiliations:** 1 Exercise Sciences Department, Brigham Young University, Provo, UT USA; 2 Mechanical Engineering Department, Brigham Young University, Provo, UT USA

**Keywords:** Doming, Short foot exercise, Toe flexion, Intrinsic foot muscles, Extrinsic foot muscles

## Abstract

**Background:**

Evaluating the strength of the small muscles of the foot may be useful in a variety of clinical applications but is challenging from a methodology standpoint. Previous efforts have focused primarily on the functional movement of toe flexion, but clear methodology guidelines are lacking. A novel foot doming test has also been proposed, but not fully evaluated. The purposes of the present study were to assess the repeatability and comparability of several functional foot strength assessment techniques.

**Methods:**

Forty healthy volunteers were evaluated across two testing days, with a two-week doming motion practice period between them. Seven different measurements were taken using a custom toe flexion dynamometer (seated), custom doming dynamometer (standing), and a pressure mat (standing). Measurements from the doming dynamometer were evaluated for reliability (ICCs) and a learning effect (paired t-tests), while measurements from the toe flexion dynamometer and pressure mat were evaluated for reliability and comparability (correlations). Electromyography was also used to descriptively assess the extent of muscle isolation in all measurements.

**Results:**

Doming showed excellent within-session reliability (ICCs > 0.944), but a clear learning effect was present, with strength (*p* < 0.001) and muscle activity increasing between sessions. Both intrinsic and extrinsic muscles were engaged during this test. All toe flexion tests also showed excellent reliability (ICCs > 0.945). Seated toe flexion tests using the dynamometer were moderately correlated to standing toe flexion tests on a pressure mat (r > 0.54); however, there were some differences in muscle activity. The former may better isolate the toe flexors, while the latter appeared to be more functional for many pathologies. On the pressure mat, reciprocal motion appeared to display slightly greater forces and reliability than isolated toe flexion.

**Conclusions:**

This study further refines potential methodology for foot strength testing. These devices and protocols can be duplicated in the clinic to evaluate and monitor rehabilitation progress in clinical populations associated with foot muscle weakness.

## Background

The evaluation of foot muscle strength may be important in a variety of clinical diagnostic and rehabilitation applications [1]. For instance, diseases such as diabetes [2] and Charcot-Marie-Tooth [3] contribute to foot weakness and altered foot neuromuscular activation [4]. Foot muscle weakness is also associated with a wide range of foot deformities and foot pain (e.g. [5–10]). Recent research has shown that foot muscle weakness may be associated with increased fall risk in the elderly due to the muscles’ role in stabilization and balance [11, 12]. Emerging evidence also suggests that targeted foot strengthening exercises may benefit those with foot pathologies [13, 14]. The ability to accurately assess foot strength may help clinicians and researchers identify weakness and monitor rehabilitation progress.

While robust methods for evaluating the strength of muscle groups crossing the major body joints is commonplace, evaluating the strength of the small foot muscles is challenging. In particular, the intrinsic foot muscles contribute to many of the same actions as larger extrinsic lower limb muscles, making it difficult to isolate their effects. Previous efforts have focused primarily on the functional movement of toe flexion, which targets a number of the smaller foot muscles (although still a combination of intrinsic and extrinsic muscles). Toe flexor strength methodology has relied on hand held dynamometry [9, 15, 16] or more qualitative tests such as the Paper Grip Test or Intrinsic Positive Test [1, 17–20]. These methods contain substantial subjective components and may thus be limited in reliability. Mickle et al. [12, 21] suggested exerting downward force on a pressure mat, creating a quantitative measurement system which could also separate the hallux from the rest of the phalanges during the same functional movement. Members of our research group recently presented a custom built toe flexion device wherein participants grip a small bar with their toes and pull against a force transducer [22]. These latter two options both appear promising from a reliability standpoint, but could elicit different muscular actions. A functionality comparison between them may help clarify their utility in strength assessments.

In our previous paper [22], we also described a novel method for assessing the strength of a doming, or short-foot [23], exercise. This movement involves contracting the quadratus plantae and other muscles crossing the midfoot to raise the medial longitudinal arch (MLA) and thus “shorten” the foot. This movement has been used as a rehabilitation tool with a goal of engaging and strengthening the intrinsic foot muscles [24], but accompanying strength assessments have not been published. Our doming device used a force transducer connected to a padded cuff placed over the dorsal foot to measure upwards MLA force. We tested within session reliability, which was quite good [22]. However, while toe flexion is somewhat habitual for most adults, doming can be an awkward movement that may require training to achieve a reliable measurement across days. The possibility of a learning effect has not been assessed.

The overall purpose of the present study was to test the repeatability and comparability of the aforementioned methods for assessing foot muscle strength. For toe flexion, our main goal was comparative, to determine whether toe flexion on a pressure mat and toe flexion on our custom-built device targeted similar muscle functions. For doming, our main goal was to assess reliability across days and evaluate the extent of a potential learning effect. For both movements, we also sought to preliminarily assess the extent to which participants were isolating the targeted muscles through the use of surface electromyography (EMG). Robust, reliable, and targeted methods of assessing foot strength may open new research opportunities to understand foot function and new clinical opportunities to affect this function.

## Methods

### Participants

Forty healthy participants (20 M, 20 F) were recruited from Brigham Young University’s campus. Sample size was calculated using Gpower software (version 3.1.9.2) and based on estimates of differences in doming as well as correlations between toe flexion tests. The former was estimated in part from a previous study [22], doubling the inter-day doming differences to account for training. This yielded an effect size of 0.5; with 80% power and α = 0.05, a sample size of 34 was estimated. Similarly, detecting a correlation effect size of r = 0.5 with the same power yielded a sample size estimate of 29. 40 participants were chosen to provide additional power.

To ensure that all participants were healthy and free from foot pain, participants were asked if they had any orthopedic injuries or impairments that might affect foot function and strength, with affirmative responses excluded. All participants were volunteers and signed consent forms approved by the Brigham Young University institutional review board in compliance with the Declaration of Helsinki. Sample demographics were: height = 172.2 ± 9.8 cm, weight = 72.3 ± 13.1 kg, age = 25.3 ± 6.2 yrs.

### Strength tests

Five strength testing protocols were performed on three different apparatus. For analysis, this resulted in a total of seven assessed strength measurements: 1) Great Toe Flexion (GTF), 2) Lateral Toe Flexion (LTF), 3) Great Toe Pressure Isolated (GTP_I_), 4) Lateral Toe Pressure Isolated (LTP_I_), 5) Great Toe Pressure Reciprocal (GTP_R_), 6) Lateral Toe Pressure Reciprocal (LTP_R_), and 7) Doming (DOM).

**Fig. 1:**
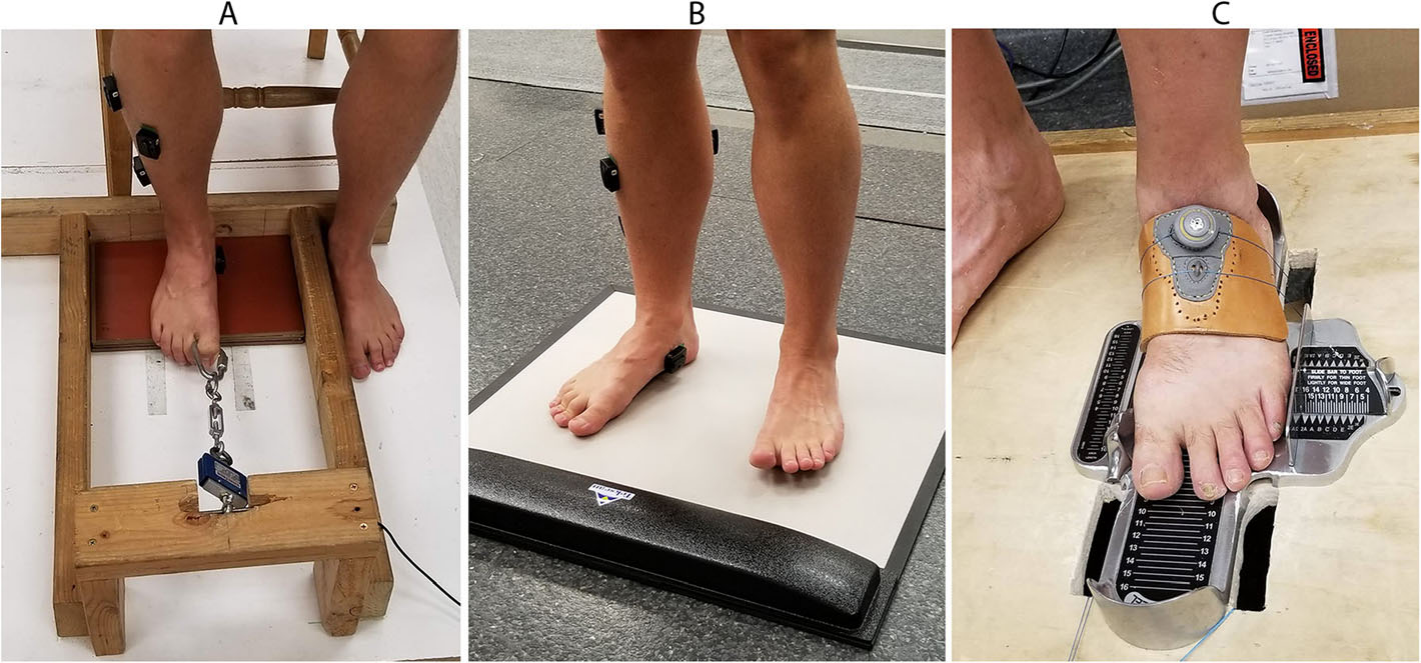
Three strength testing apparatus were used to create seven strength assessments. **a**) Toe flexion device (GTF and LTF), **b**) Pressure mat (GTP_I_, LTP_I_, GTP_R_, and LTP_R_), **c**) Doming (DOM) – the force transducer is in tension underneath the wooden platform

Both GTF and LTF used the same device, which was described in detail previously [22]. Briefly, participants sat in a chair with their knees at a 90° angle and testing foot flat on a board that spanned from the heel to 1st metatarsal head. Participants then gripped a carabiner with the great toe (GTF) or a T-bar with the 2nd and 3rd toes (LTF) and pulled on a cable attached to a force transducer (model SMT1–22, Interface, inc. Scottsdale AR, USA) (Fig. 1a). One modification was made to the previous design - the transducer was raised slightly to reduce the toe flexion angle required to hold the carabiner T-bar. A turnbuckle attached between the transducer and carabiner was adjusted until a baseline tension between 1.5–3.0 N was achieved in a stable, but not exerted, position. Upon a signal from the investigator, participants pulled with maximal effort sustained over a three second period.

GTP_I_, LTP_I_, GTP_R_, and LTP_R_ were all performed on a pressure mat (HR Mat, Tekscan inc., Boston MA, USA) in a manner patterned after Mickel et al. [21]. GTP_I_ and LTP_I_ came from the same tests, as did GTP_R_ and LTP_R_. For the former, participants stood in the center of the pressure mat and were asked to isolate and press the hallux and toes of the testing foot as hard as they could into the pressure mat, holding for three seconds. Total force under each foot was monitored during the test, with no more than 60% of body weight allowed on the testing foot. The reciprocal tests were identical to the isolated tests, but the contralateral toes were extended concurrently with flexion of the testing toes (Fig. 1b). For both tests, force under the great toe and lateral toes was separated during analysis.

The device used for DOM was modified more extensively from that previously described [22]. The transducer (Model LC101–100, Omegadyne, inc., Stamford CT, USA) was moved from above the foot to below it, creating a pulling action with the transducer in tension instead of compressive. This was done to alleviate any possibility of misalignment between foot motion and force transducer axis. Participants stood on a small wooden box, placing the testing foot in a brannock device. A molded plastic cuff was then placed over the dorsal foot. Wires secured the cuff to the transducer inside the box and were tightened using a turnbuckle to a pre-load of 4.5 N - 5.5 N. Doming was accomplished by shortening the foot and pushing the arch upwards against resistance, exerting maximally for three seconds. Instructions were given to slide the ball of the foot towards the heel without curling the toes. Participants were given several practice trials prior to collection. Note that following study completion, the molded plastic cuff was replaced with a leather cuff, and the turnbuckle was replaced with a Boa enclosure system (Boa Technology inc., Denver, CO, USA), which improved comfort and ease of use (Fig. 1c).

### Protocol

Data collection was performed on two separate days, two weeks apart. The strength testing protocol for both days was identical, with one exception. On the first day, a second session was performed on the DOM test, to better assess acute learning effects. Height, weight and foot dominance were also documented on the first day. Testing was performed on the dominant foot only, which was determined by foot preference in 2 out of 3 activities (step-up, ball kick, and response to push).

**Fig. 2:**
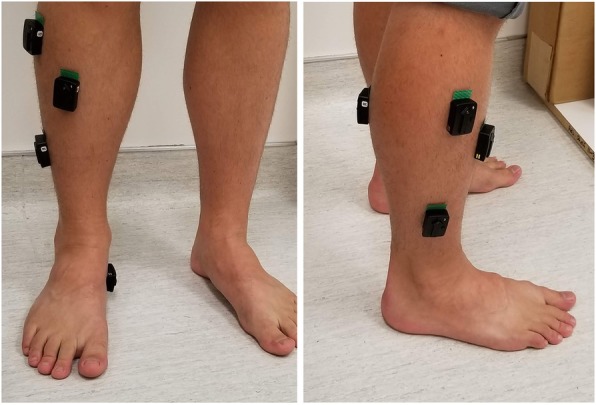
EMG electrode placement. Surface electrodes were placed over the tibialis anterior (TA), fibularis longus (FL), fibularis brevis (FB), abductor hallucis (AH), and medial gastrocnemius (GS)

Prior to strength testing, five wireless, bipolar, Ag/AgCl surface electrodes (Trigno, Delsys Inc., Boston, MA, USA) were affixed to the skin surface of the dominant foot, using double-sided tape. Locations corresponded to the muscle bellies of the medial gastrocnemius (GS), fibularis longus (FL), fibularis brevis (FB), tibialis anterior (TA), and abductor hallucis (AH) (Fig. 2). Two functional movements were then performed to elicit near-maximal muscle contractions and establish reference values for EMG activation. The first was a single-leg calf raise (with handheld assist), held for three seconds. This movement was used as a reference for the GS, FL, FB, and AH muscles. In the second movement, participants balanced on both heels, dorsiflexing their ankles and raising their toes off the ground, again holding for three seconds. This was used as a reference for the TA muscle. EMG was collected in all tests at 1926 Hz.

Three trials of each of the five strength tests were performed barefoot on the dominant foot. The order of the five tests was randomized using a practical two-tiered approach (i.e. split-plot design). First, the order of the three apparatus were randomized (doming device, toe flexion device, and pressure mat); next the order of the individual tests performed at each device were randomized (e.g. GTF and LTF). For each trial, participants were instructed to exert maximal effort and hold for three seconds. Data from the devices using force transducers (GTF, LTF, and DOM) were collected at 1000 Hz using LabView (National Instruments DAQ 9171, Austin TX USA), while data from the pressure mat was collected at 100 Hz through the manufacturer’s software interface.

After the first day, participants were given instructions to practice the doming motion prior to the second day. A minimum of 10 exercise bouts were required during the two weeks between days, each bout consisting of 2 sets of 10 contractions. Investigators contacted the participants on a daily basis to provide reminders. While the second test day was instituted primarily to assess potential learning effects for the DOM tests, all tests were repeated for consistency, and the protocol for day two was therefore identical to day one (with the exception of the second DOM session).

### Data processing

**Fig. 3:**
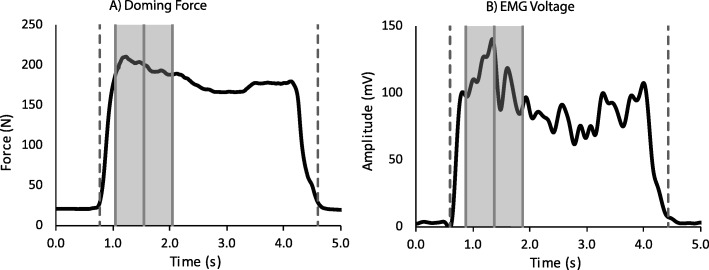
Example doming force (**a**) and EMG (**b**) plots. Force and EMG were extracted over a 1-s time window (*shaded area*). The onset and termination of force are marked with *dotted lines*, which were aligned with the same events in the EMG signal for synchronization. Mean EMG magnitude was calculated over the same time window

Data from the force transducers was analyzed in custom LabView software (National Instruments, Austin TX USA). Transducer force was plotted over time with a movable one-second window designated on the graph. The program automatically placed the window over the region with the highest one-second average force (Fig. 3a). However, if this did not exhibit a plateau-like shape, the operator moved the window to the most suitable location, visually balancing high force with low variability. The extracted one-second average force metric was used for statistical analysis.

Data from the pressure mat was initially processed in Tekscan’s FootMat software (v. 7.1). Masks were manually drawn around the great toe and lateral toes, and total force under each of these areas, expressed as a percentage of total force, was exported. This was then multiplied by body mass to convert to force units. Region forces were then imported into the same LabView software and analyzed in the same manner as the transducer forces.

EMG signals were also processed in LabView. All signals were zero-offset, low pass filtered (350 Hz 4th order Butterworth), rectified, and high pass filtered (5 Hz 4th order Butterworth) to get a linear envelope. EMG signals were then time-synchronized with their associated force profiles (Fig. 3b), and the average envelope amplitudes during the same one-second windows were extracted. For the force transducers, the electrodes were collected through the same software, allowing for direct software synchronization. For the pressure mat, however, EMG and pressure were collected separately, initiated by a verbal command. Synchronization was fine-tuned afterwards by manually aligning the onset of muscle activation with the increase in force production. For presentation, EMG amplitudes were normalized to their associated reference near-maximal contractions and expressed as percentages.

### Statistical analysis

Repeatability of all tests was assessed using intraclass correlation coefficients (ICC) with random participants and fixed raters for absolute agreement as well as Standard Error of the Measurement (SEm). These were calculated across the three trials for each session independently. Mean differences between each day were compared using paired t-tests, except for DOM, which contained three total sessions. For this, a one-way repeated measures ANOVA was used, followed by LSD post-hoc pairwise comparisons. Comparability of toe flexion strength between the toe flexion device and the pressure mat tests was done using Pearson correlation coefficients. Significance for all tests was set at α = 0.05. Descriptive statistics were used to evaluate muscle activation during each test. For DOM, EMG data from all three sessions are presented, while for the other tests only the data from the second day tests are presented.

## Results

All devices showed excellent within-session repeatability (Table 1), with ICC values between 0.945 (GTP_I_, day 1) and 0.986 (LTP_R_, day 2). ICCs for all tests increased slightly between day 1 and day 2.

**Table 1:** Measurement repeatability

Measure	Day 1		Day 2
DOM	S1: 0.944	S2: 0.974	0.973
	(0.903–0.969)	(0.953–0.986)	(0.956–0.984)
GTF	0.961		0.984
	(0.931–0.979)		(0.974–0.991)
LTF	0.965		0.982
	(0.942–0.980)		(0.969–0.990)
GTP_I_	0.945		0.974
	(0.913–0.967)		(0.958–0.984)
LTP_I_	0.949		0.962
	(0.918–0.969)		(0.939–0.977)
GTP_R_	0.952		0.983
	(0.923–0.971)		(0.972–0.990)
LTP_R_	0.952		0.986
	(0.923–0.971)		(0.978–0.992)

Intraclass Correlation Coefficients are shown along with associated 95% Confidence Intervals in parentheses. All measures contained one set of three trials on both days, with the exception of DOM, which included two sets on day 1 (S1 and S2)

While the mean force values (Table 2) for all tests also increased slightly between day 1 and day 2, only in DOM did this reach a statistically significant difference. Pairwise comparisons among the three DOM sets showed a significant change between day 2 and both sets of day 1 (*p* < 0.001), but no difference between the two sets on day 1 (*p* = 0.111).

**Table 2:** Raw force data (mean ± standard deviation) for each measure, expressed in Newtons. *P*-values for paired t-tests between days are shown. For DOM, the *p*-value is from a full one-way ANOVA across all three sets. Pairwise comparisons are displayed below the table

Measure (N)	Day 1		Day 2	*P*-value
DOM	S1: 117.9 ± 72.4	S2: 131.0 ± 72.0	164.2 ± 86.0	< 0.001 (ANOVA)*
GTF	50.0 ± 22.0		53.1 ± 27.0	0.193
LTF	41.9 ± 18.5		46.8 ± 24.3	0.517
GTP_I_	51.6 ± 32.6		56.0 ± 35.7	0.137
LTP_I_	86.4 ± 41.3		93.4 ± 45.5	0.121
GTP_R_	57.1 ± 32.4		62.6 ± 43.9	0.142
LTP_R_	95.7 ± 42.5		102.3 ± 57.0	0.231

* Pairwise comparisons: p = 0.111 for day 1, set 1 vs. 2; *p* = 0.001 for day 1 set 2 vs day 2; p < 0.001 for day 1 set 1 vs day 2

**Fig. 4:**
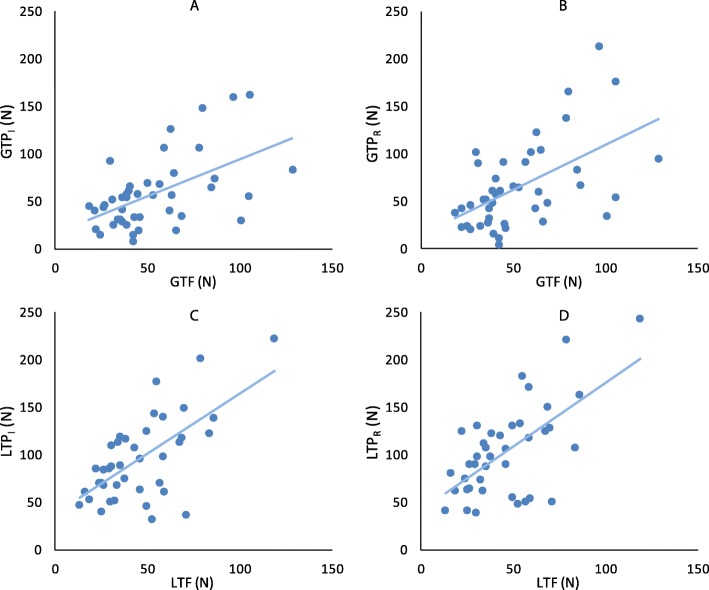
Correlations between the toe flexion device and pressure mat. Left column = isolated tests, right column = reciprocal tests; top row = great toe, bottom row = lateral toe: **a**) GTP_I_ vs GTF, **b**) GTP_R_ vs GTF, **c**) LTP_I_ vs LTF, **d**) LTP_R_ vs LTF. See Methods for specific acronym names

Both toe flexion tests were significantly correlated with their respective pressure mat tests (p < 0.001) (Fig. 4). GTF was moderately correlated with both GTP_I_ (r = 0.54) and GTP_R_ (r = 0.55), while LTF was slightly more, but still moderately, correlated with both LTP_I_ (r = 0.65) and LTP_R_ (r = 0.64).

**Fig. 5:**
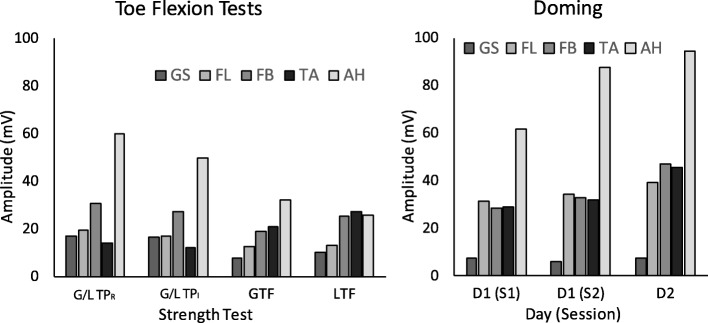
Mean muscle EMG activation levels. Comparisons among all toe flexion tests, day 2 (*left*), and among the three sessions of doming (*right*)

Mean EMG activation levels across tasks varied by muscle (Fig. 5). GS EMG activity was small during all tests, but largest in the pressure mat tests (~ 17% max). TA activity ranged from 12% (GTP_I_ / LTP_I_) to 45% (DOM) max. FL and FB had comparable activation levels in DOM (~ 36% max), while FB activity was slightly greater than FL across all toe flexion tasks (~ 26% vs ~ 16% max). AH activity ranged from 25% (LTF) up to 94% (DOM) max. In DOM, all muscle activations appeared to increase across the three sets.

## Discussion

The purposes of this study were to assess repeatability and comparability of several functional foot strength assessment techniques. For discussion, doming is separated from the various toe flexion tests.

### Toe flexion

Both the toe flexion device and the pressure mat appear to be reliable tools for assessing toe flexor muscle strength, albeit with some functional differences between them. Within session repeatability was high for both devices and both were moderately correlated to each other. Notably, the correlation plots were more dispersed at higher forces (Fig. 4), suggesting that the execution strategies diverged between devices as force increased. Also, the forces under the hallux were higher than the lateral toes on the toe flexion device, while the reverse was true for the pressure mat. Correlation coefficients between devices were also higher for the lateral toes than the hallux. It may be that the instruction to flex all toes at one time on the pressure mat resulted in a strategy that unequally focused on the lateral toes. EMG also suggests that foot musculature was being activated to a slightly different degree between devices. In particular, the pressure mat engaged the AH more. This is consistent with the greater lateral toe pressure, and is due in part to the standing posture [23]. Because of the need for stabilization and balance when standing, the pressure mat may represent a more functional movement, while the toe flexion device likely better isolates the toe flexor muscles. The latter may be more preferable when assessing specific changes in muscle strength, due to fewer compensatory strategies. However, participants with compromised distal motor control, deformities, or sensation loss (e.g. diabetic neuropathy) may find it easier to press downward on the pressure mat than to grip the toe flexion device.

When using a pressure mat to assess toe flexion, a number of factors suggest that reciprocal motion may be preferable over isolated motion. The reciprocal test had slightly higher ICCs as well as higher mean forces. It also appeared to be subjectively more comfortable, as the reciprocal motion helped the participant maintain a stable posture with even weight distribution between feet, avoiding a forward lean. This is not surprising given the heavy use of reciprocal motion in the lower extremity during walking, running, and other functional movements – the nervous system appears to be wired for reciprocal motion in the lower extremity, in contrast with more mirrored movements in the upper extremity [25, 26].

Our toe flexion force magnitudes differ from previously reported values, most likely due to methodological differences. In our own previous study [22], we reported slightly higher toe flexion forces than the current study, perhaps due to the subtle design modifications. Most other studies reported substantially higher values than ours, ranging from 60 to 130 N [16, 27], likely due to different positioning (e.g. standing or lying supine), participant samples, and equipment. We chose our testing methodology in a specific effort to isolate toe flexor muscles, and the lower force values may represent successful isolation. The previous toe flexion dynamometry studies [16, 27] are actually closer in magnitude to our pressure mat values, which may be in part due to the standing posture. Our pressure protocol was based on Mickle et al. [13]; however, we showed lower pressures under the hallux (18% BW compared to 8% BW) and higher pressures under the lateral toes (11% BW compared to 14% BW). The main difference between the studies was that Mickle used separate tests for the hallux and lateral toes. We chose to capture force using a single test in an attempt to reduce the involvement of compensatory or stabilizing muscles that may be engaged when trying to isolate the great toe or lateral toes. For example, hallux extension or hindfoot inversion could increase lateral toe force by unloading the medial side of the foot. Both ours and Mickle’s values are slightly higher than Menz et al. [19], who employed the paper grip test over a similar pressure mat.

### Doming

Due to its novelty, there is little previous research with which to compare our doming strength data. In contrast to our toe flexion tests, our doming values are slightly higher than our previous study [22], but likely for similar reasons (modifications made to the device interface). While within session repeatability was excellent in both studies, DOM showed a clear learning effect, as forces and ICCs both increased between day 1 and day 2. EMG reveals some insight into this learning. With the exception of the GS, the activity of the other muscles mirrored the force, increasing with each successive set and motion familiarity. The AH in particular showed a large increase, even between the two sets on day 1. The AH is the primary intrinsic foot muscle responsible for MLA rise, and it is not surprising that increased familiarity with the awkward doming movement increased its role as well as DOM strength values. While the FL and FB do not directly contribute to MLA rise, their activity also increased, likely due to the increased need for stabilization to control foot inversion that accompanies MLA rise. Accustomization appeared to happen even between sessions within a day – as evidenced by the combination of higher (although not significant) mean forces, ICCs, and muscle activity. The amount of practice required to become fully accustomed to the motion is not clear from this study, but it is clear that some amount of practice should be employed for clinical utility.

While the GS was mostly inactive during the doming motion, the TA was clearly used by the participants, and its activation also appeared to increase with familiarity. Both the TA and the tibialis posterior (TP) cross the midfoot and are extrinsic facilitators of MLA rise. While it is difficult to fully isolate the AH from the TA and TP, the AH is clearly active during the doming movement, particularly when it is performed standing [23]. Doming has been proposed as a rehabilitation exercise to strengthen intrinsic foot muscles, in part because it engages the AH more than toe flexion does (ref 23 here too). However, the extrinsic TA and TP muscles can overpower or overcompensate for weak intrinsic muscles [28], causing an existing muscle imbalance to persist even after doming training. Future studies should focus on training strategies that isolate the AH from the TA and TP.

### Limitations and future directions

The main study limitations revolved around the use of surface EMG to assess muscle isolation. This yielded some insights, but could not fully assess intrinsic muscle activity; this would require fine-wire EMG. In addition, EMG activity was normalized to a functional maximum. This was chosen because individual muscle maximums are difficult to obtain for the feet. Jung et al. [23, 29] used a manual muscle test to represent maximum AH contraction – this adds tester variability and may be an unfamiliar movement for many participants, but should be considered in future work. We also did not fully assess the repeatability of the strength tests between sessions or between raters; however, this was performed previously [22]. We also chose not to normalize the force values to give readers a sense of the strength magnitudes and normalization was not needed for our within-subject comparisons. However, appropriate normalizations (e.g. to body weight) may need to be investigated, for example when comparing strength changes among participants (i.e. between-within designs). Another future direction is to make use of the variability in the force-time waveform to investigate force steadiness (e.g. [30, 31]), which may provide insight into motor dysfunction.

## Conclusions

Overall, this study further established several tools that can be used to quantify foot muscle strength. For toe flexion strength evaluation, our recommendation for choosing between dynamometry and pressure mat tests depends on the clinical application. The toe flexion device is likely better for isolation and assessing changes over time, while the pressure test is more functional and easier for some populations to perform. For the latter, we recommend using a reciprocal motion between limbs. Practically, the toe flexion device can be constructed inexpensively, but requires a dedicated apparatus and software. Pressure mats are often used clinically for gait and posture assessments, however, foot strength analysis requires some custom post-processing. For doming, our novel test could be duplicated in the clinic, but would also require a separate apparatus and software. We are currently working on an integrated design that combines toe flexion and doming in a single device. We recommend that instruction and practice of the doming motion be provided to patients prior to initial testing.

## Data Availability

The data generated by this study will be available from the corresponding author on reasonable request.
